# Fuel

**Published:** 1859-10

**Authors:** 


### HALL'S JOURNAL OF HEALTH.

OUR LEGITIMATE SCOPE IS ALMOST BOUNDLESS: FOR WHATEVER BEGETS PLEASURABLE AND HARMLESS FEELINGS, PROMOTES HEALTH; AND WHATEVER INDUCES DISAGREEABLE SENSATIONS, ENGENDERS DISEASE.

*We aim to show how Disease may be avoided, and that it is best, when sickness comes, to take no Medicine without consulting an educated Physician.*

VOL. VI.] OCTOBER, 1859. [No. 10.

### FUEL.

Wood is the healthiest, because it contains a large amount of oxygen; coal has none, hence, in burning it, the oxygen necessary for its combustion must be supplied from the air of the room, leaving it "close," oppressive.

A coal fire will go out unless it has a constant and large supply of air, while wood, with comparatively little, having a large supply within itself, turns to "live" coals.

Close-grained heavy woods, like hickory and oak, give out the most heat; while pine and poplar, being open-grained, heat up the quickest.

The value of fuel as a heating material, is determined by the amount of water which a pound will raise to a given temperature; thus, one pound of wood will convert forty pounds of ice to boiling water, while a pound of coal will thus heat near eighty pounds of ice cold water; hence, pound for pound, coal is as good again as wood for mere heating purposes, and wood is as good again as peat, which is the product of sedges, seeds, rushes, mosses, &c.

But, if a ton of coal, that is, twenty-eight bushels, or twenty-two hundred and forty pounds, costs five dollars, it is about equal to the best wood at two dollars and a quarter a cord. Coal, at twelve dollars and a half a ton, is as cheap as wood at five dollars and a half a cord. It would be more equitable, if wood was dry, to sell it by the pound. Such is the custom in France.

A certain amount of wood, giving out a hundred degrees of heat, the relative value in this country is as follows:

| | | | |
|---|---|---|---|
| Shell-bark Hickory, - | 100 | Apple Tree, - - - - | 70 |
| Pig-nut Hickory, - - | 95 | Ash, White, - - - - | 77 |
| White Oak, - - - - | 84 | Beach, White, - - - | 65 |
| White Ash, - - - - | 77 | Birch, Black, - - - | 62 |
| Dog Wood, - - - - | 75 | Birch, White, - - - | 48 |
| Scrub Oak, - - - - | 73 | Butter-Nut, - - - - | 51 |
| White Hazel, - - - | 72 | Cedar, Red, - - - - | 56 |
| Apple Tree, - - - - | 70 | Cherry, Wild, - - - | 55 |
| Red Oak, - - - - | 69 | Chesnut, - - - - - | 52 |
| White Beech, - - - | 65 | Dog Wood, - - - - | 75 |
| Black Walnut, - - - | 65 | Elm, White, - - - - | 58 |
| Black Birch, - - - - | 62 | Hazel, White - - - | 72 |
| Yellow Oak, - - - - | 60 | Hickory, Pig-Nut, - - | 95 |
| Hard Maple, - - - - | 59 | Hickory, Shell Bark - | 100 |
| White Elm, - - - . | 58 | Maple, Hard, - - - | 59 |
| Red Cedar, - - - - | 56 | Oak, Red, - - - - | 69 |
| Wild Cherry, - - - | 55 | Oak, Scrub, - - - - | 73 |
| Yellow Pine, - - - | 54 | Oak, White, - . - - | 84 |
| Chesnut, - - - - - | 52 | Oak, Yellow, - - - | 60 |
| Yellow Poplar, - - - | 52 | Pine, White, - - - | 42 |
| Butter Nut, - - - - | 51 | Pine, Yellow, - - - | 54 |
| White Birch, - - - | 48 | Poplar, Yellow, - - | 52 |
| White Pine, - - - - | 42 | Walnut, Black, - - - | 65 |

A cord of wood measures eight feet long, four feet broad, and four feet high. A short ton (2,000 lbs.) of anthracite coal will fill a box or bin, level full, containing thirty-four and a half feet cubical. A legal ton calls for forty feet. The length, breadth, and depth of a box or bin, multiplied together, and the product divided by two thousand, or twenty-two hundred and forty, gives the contents in tons.

One pound of coal will do as much work as a strong man in a given time.

An ocean steamer consumes five thousand pounds of coal in one hour, hence, it would require five thousand men to move a steamship as fast and far as it usually moves in an hour.

A steamship will consume more coal in one day than a large family will in a year, or than will supply an ordinary fire for ten years. But, a kind and wise Providence has, with a a magnificent liberality acted towards us, in that there is more coal in the United States, by many millions of tons, than there is in Great Britain, France, Spain, Portugal, Belgium, and British North America together—as far as yet discovered. Missouri alone, is estimated to be able to furnish a hundred millions of tons of coal every year for more than a thousand years to come. London consumes a million and a half of tons a year; hence, the State of Missouri alone could supply more than sixty such cities as London every year with the coal necessary to warm all their dwellings, and drive all their machinery.

And what is coal, that lights our streets and warms our dwellings, and is the power behind the throne of—steam? It is preserved sunshine; for, without sunshine, there can be no vegetation, and coal is the product of vegetation; that is, vegetable matter, subject to great pressure, is thereby, in the long process of ages, converted into coal—just as we see it. Thus, the sunshine, in the shape of its caloric, its heat, of the myriads of ages which may have passed when man was not, was not lost, but was gathered, as it were, and condensed and preserved beforehand, that when he came, he might abundantly use it to happify and elevate the race, and develope its power; so that nothing is wasted, not even the sunshine, and "God is Love."

